# Association between Ambient Air Pollution and Asthma Prevalence in Different Population Groups Residing in Eastern Texas, USA

**DOI:** 10.3390/ijerph13040378

**Published:** 2016-03-29

**Authors:** Amit Kr. Gorai, Paul B. Tchounwou, Francis Tuluri

**Affiliations:** 1 Department of Mining Engineering, National Institute of Technology, Rourkela, Odisha 769008, India; 2 NIH/NIMHD RCMI Center for Environmental Health, College of Science, Engineering and Technology, Jackson State University, Jackson, MS 39217, USA; 3 Department of Industrial System and Technology, Jackson State University, Jackson, MS 39217, USA; francis.tuluri@jsums.edu

**Keywords:** air pollution, asthma, age, gender, race, Texas, USA

## Abstract

Air pollution has been an on-going research focus due to its detrimental impact on human health. However, its specific effects on asthma prevalence in different age groups, genders and races are not well understood. Thus, the present study was designed to examine the association between selected air pollutants and asthma prevalence in different population groups during 2010 in the eastern part of Texas, USA.The pollutants considered were particulate matter (PM_2.5_ with an aerodynamic diameter less than 2.5 micrometers) and surface ozone. The population groups were categorized based on age, gender, and race. County-wise asthma hospital discharge data for different age, gender, and racial groups were obtained from Texas Asthma Control Program, Office of Surveillance, Evaluation and Research, Texas Department of State Health Services. The annual means of the air pollutants were obtained from the United States Environmental Protection Agency (U.S. EPA)’s air quality system data mart program. Pearson correlation analyzes were conducted to examine the relationship between the annual mean concentrations of pollutants and asthma discharge rates (ADR) for different age groups, genders, and races. The results reveal that there is no significant association or relationship between ADR and exposure of air pollutants (PM_2.5_, and O_3_). The study results showed a positive correlation between PM_2.5_ and ADR and a negative correlation between ADR and ozone in most of the cases. These correlations were not statistically significant, and can be better explained by considering the local weather conditions. The research findings facilitate identification of hotspots for controlling the most affected populations from further environmental exposure to air pollution, and for preventing or reducing the health impacts.

## 1. Introduction

Air pollution is one of the major environmental threats to urban populations [1]. It can be traced back to the industrialization-urbanization era started in the 19th century. Air pollution has been reported to cause adverse health impacts on people of all races, ages, and genders in every country including the USA. The past studies revealed that exposure to air pollution has more serious health effects such as reduced life expectancy, increased daily mortality and hospital admissions, birth outcomes, and asthma [2] than previously thought. Künzli *et al.* estimated that 40,000 deaths annually or 6% of total mortality amongst adults aged 30+years were due to ambient air pollution in Austria, Switzerland, and France [3]. In another study, Valent *et al.*, reported that some 13,000 deaths annually of infants (ages 0–4) were attributed to outdoor air pollution [4], making it a second environmental burden of disease only to physical injuries across Europe. Over the last two decades, many epidemiologic studies have confirmed the positive association between air pollution and mortality [5,6,7,8,9,10]. To analyze these effects, air pollution epidemiology studies with useful assessment techniques have been implemented to detect the small increase in risk due to increase in the background pollution level. Additionally, the studies have consistently showed that exposure to common air pollutants is linked to many adverse health outcomes including respiratory (chronic obstructive pulmonary disease), asthma, and lung cancer [11,12,13,14,15,16] and cardiovascular diseases [12,17,18,19,20,21].

Airborne pollutants influence the symptoms of asthma in patients [22,23,24]. Asthma is a burden, not only in developing countries, but also in developed countries, with significant impacts on public health and economics. As per the 2011 National Health Survey Data of United States, asthma is identified as one of the most prevalent chronic diseases among U.S. children. A total of 10.5 million (14%) U.S. children have been diagnosed with asthma [25]. Asthma is a serious and sometimes life-threatening chronic respiratory disease that affects almost 25 million Americans and costs the nation $56 billion per year [26]. In 2009, 3.3 deaths per 100,000 people were attributed to asthma, and 1.9 million asthma-related emergency department visits were recorded [27,28]. Asthma prevalence increased from 7.3% in 2001 to 8.4% in 2010 [29]. It was also revealed that children may be more susceptible to ambient air pollution than adults [30,31], and are disproportionately affected by asthma, as evidenced by higher asthma hospitalization rates for persons under age 18 [32]. These adverse health outcomes have been shown to be similar in both economically developing and developed countries [33].

Many specific epidemiologic studies have found that short-term exposure of ozone leads to adverse health effects ranging from mild respiratory function impairment to increased mortality [10,34,35,36,37]. It is also confirmed in many studies that short-term ambient ozone exposures increases the asthma emergency department (ED) visits [38,39,40,41,42] and hospitalizations [43,44,45], particularly during the warm season when ozone concentrations are higher [46,47]. Further, many epidemiologic studies have been conducted on the association between exposure to ozone with asthma exacerbations in children [48,49,50,51], but results have not always been consistent. Thus, many questions regarding the effects of air pollution remain unanswered, and overall, these effects have not been adequately quantified. Furthermore, the differences in health effects due to air pollution exposure on different races, age groups, and genders are not properly established. In various studies, results have varied widely, particularly for effects between racial groups, genders, and age groups. According to Zeka *et al.*, the impacts of air pollution was similar among races while others found significant differences for Whites and Hispanics [52], but not African Americans [53], or for African Americans but not for other races or ethnic groups [54]. Few other researchers have found the greater risk for African Americans from air pollution [55]. The evidence of significant adverse effects of air pollution on public health has led to more stringent air quality norms in many countries including the USA.

Though, current research indicates relationship between social-demographic characteristics (e.g., age, race, *etc.*) and disease [56], the underlying reasons for disparities in disease have not fully elucidated. Additionally, it is known that asthma attacks are triggered by multiple criteria pollutants, namely particulate matter (PM), ozone (O_3_), sulfur dioxide (SO_2_), nitrogen dioxide (NO_2_), and carbon monoxide (CO) [18,20,57,58,59]. However, the specific effects of these air pollutants on asthma prevalence in different age groups, genders, and races are not well understood. Thus, the aim of the present study was to examine the association between selected air pollutants (PM_2.5_ and ground-level ozone), and asthma prevalence in different population groups (based on age, gender, and race) residing in eastern part of Texas, USA using geospatial techniques.Due to an insufficient number of spatial data on other criteria air pollutants monitored in the region, the present study focused only on PM_2.5_ and ground-level ozone.

## 2. Study Area

**Figure 1:**
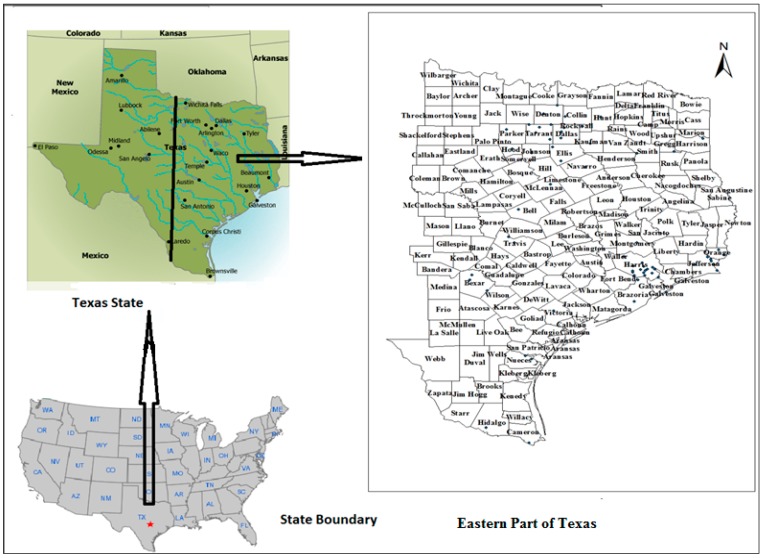
Study area map.

The eastern part of Texas State, USA (shown in the Figure 1) was selected for the proposed study. Due to the inexistence of a sufficient number of air pollution monitoring stations in the western part of the state, only eastern part was considered for the study. Texas State is located in the west-south-central region of the United States. The extent of longitude and latitude of the state are 93°31′ W to 106°38′ W and 25°50′ N to 36°30′ N, respectively. Texas is the second most populous (25,145,561), and 29th most densely populated (37.2 inhabitants per square kilometer of land area) state of the 50 United States [60]. Texas covers 696,241 square kilometers of land area and ranks as the 2nd state by size [61]. In general, the climate in Texas varies widely, from arid in the west tohumidin the east. There is significant variation in the geography from one region to another of the state. There are coastal regions, mountains, deserts and wide-open plains. In coastal regions, the weather is neither particularly hot in the summer nor particularly cold during the winter. East Texas has a humid subtropical climate typical of the southeast, occasionally interrupted by intrusions of cold air from the north.

## 3. Data

### 3.1. Asthma Data

**Figure 2:**
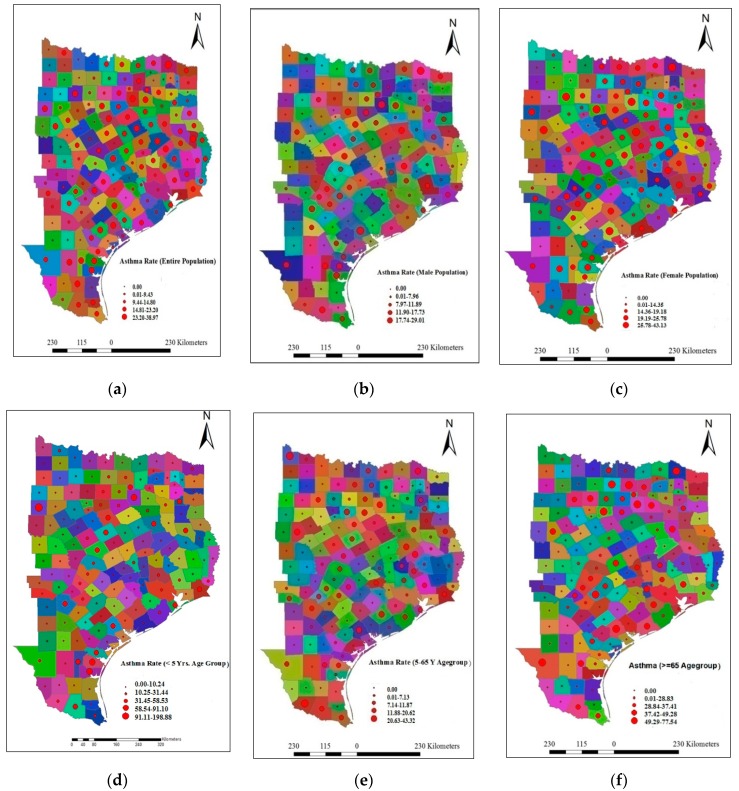

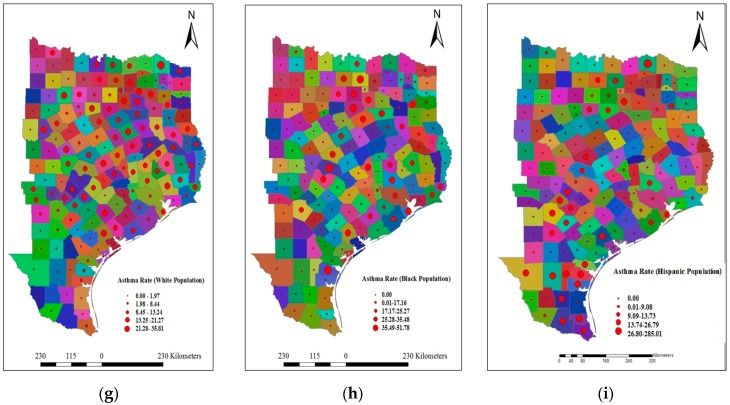
County-wise asthma rates in eastern part of Texas State (**a**) Entire Population; (**b**) Male Population; (**c**) Female Population; (**d**) <5 Year Age Group; (**e**) 5–65 Year Age Group; (**f**) >65 Age Group; (**g**) White Population; (**h**) Black Population; (**i**) Hispanic Population.

The proposed study conducted using a full year of asthma data from January 2010 to December 2010. The asthma hospital discharge numbers for different population groups (age-wise, gender-wise, and race-wise) were obtained through a personal request to Texas Asthma Control Program, Office of Surveillance, Evaluation and Research, Texas Department of State Health Services [62]. The asthma hospital discharge numbers were diagnosed as per the International Classification of Disease, Ninth Revision, Clinical Modification (ICD-9-CM) diagnosis code 493 [63]. The county-wise estimated population data were also obtained from the same office for determining the asthma rate. Asthma discharge rate (ADR) represents the number of asthma-related hospital discharges per 10,000 people for a specified period. The counties with less than 12 asthma hospital discharge cases were not reported and, thus the asthma discharge rate was considered to be zero. The county-wise asthma rate for different races (Black, White, Hispanic), age groups (<5 year, 5–65 year, and >65 year), and genders (Male, and Female) are represented in Figure 2. The reason for selecting these age groups was based on the availability of asthma data that were collected by the Texas Department of State Health Services according to these age groups.

The data shown in Figure 2 indicate that the maximum ADR for entire population group was 38.97; whereas, the ADR values for the male and female population groups were 29.01 and 43.13 respectively in 2010. The ADR values for the three age groups (<5 year, 5–65 year, and >65 year) were 198.88, 43.32, and 77.54, respectively. Similarly, the ADR values for White, Black, and Hispanic population group were recorded as 35.81, 285.01, and 51.78, respectively. Thus, the data clearly indicate that the asthma rate was higher for the female population group in comparison to the male population group. Similarly, age-wise asthma rate indicate that children are more affected than the middle-aged and older population groups. Additionally, the data confirms that Hispanic population group are more vulnerable than the White and Black population groups.

**Figure 3:**
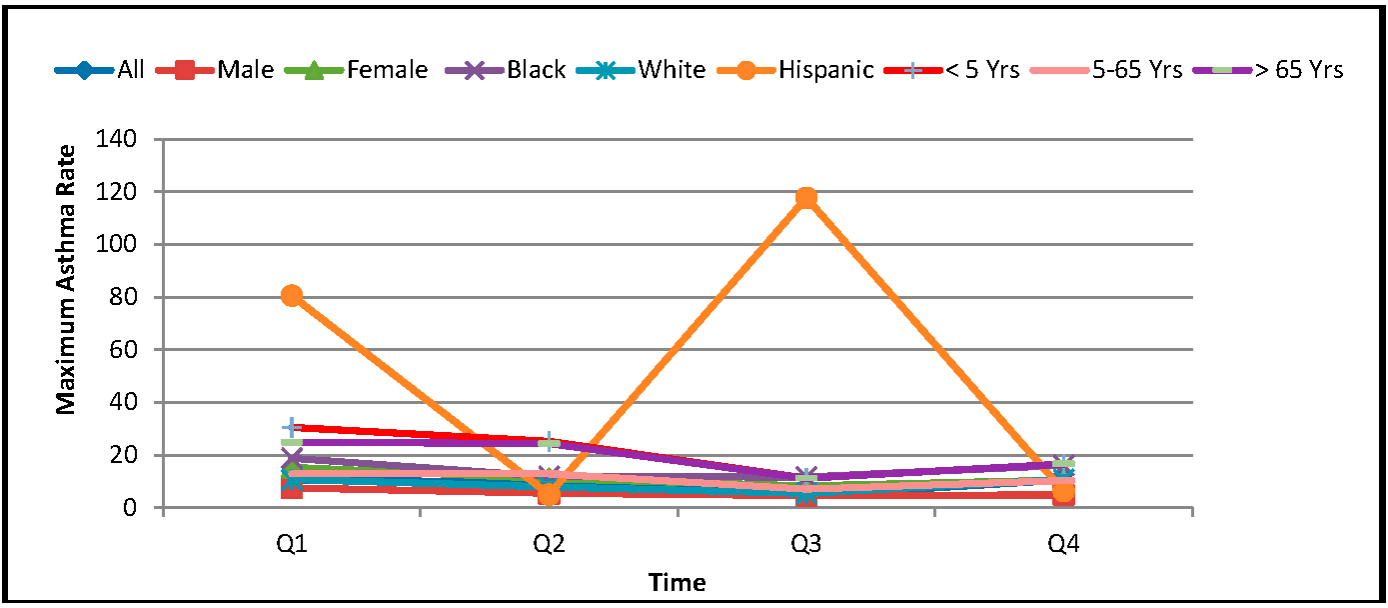
Quarterly maximum asthma discharge rate (ADR) in different groups.

The quarterly asthma discharge rate data were also calculated for seasonal study. The entire year was divided into four quarters (first quarter (Q1): January to March; second quarter (Q2): April to June; third quarter (Q3): July to September; and fourth quarter: October to December). The reason for selecting the quarters for this study was based on the fact that the asthma data was available on quarterly basis. The maximum ADR of different races (Black, White, Hispanic), age groups (<5 year, 5–65 year, and >65 year), and gender (Male, and Female) in different quarters of 2010 are represented in Figure 3. It clearly indicates that the ADR was found to be relatively high in the first quarter (Q1) for most of the groups. The ADR was found to be lowest in the third quarter (Q3) for most of the groups except the Hispanic population group. The descriptive statistics of quarterly average ADR are presented in Table 1.

### 3.2. Air Pollution Data

**Figure 4:**
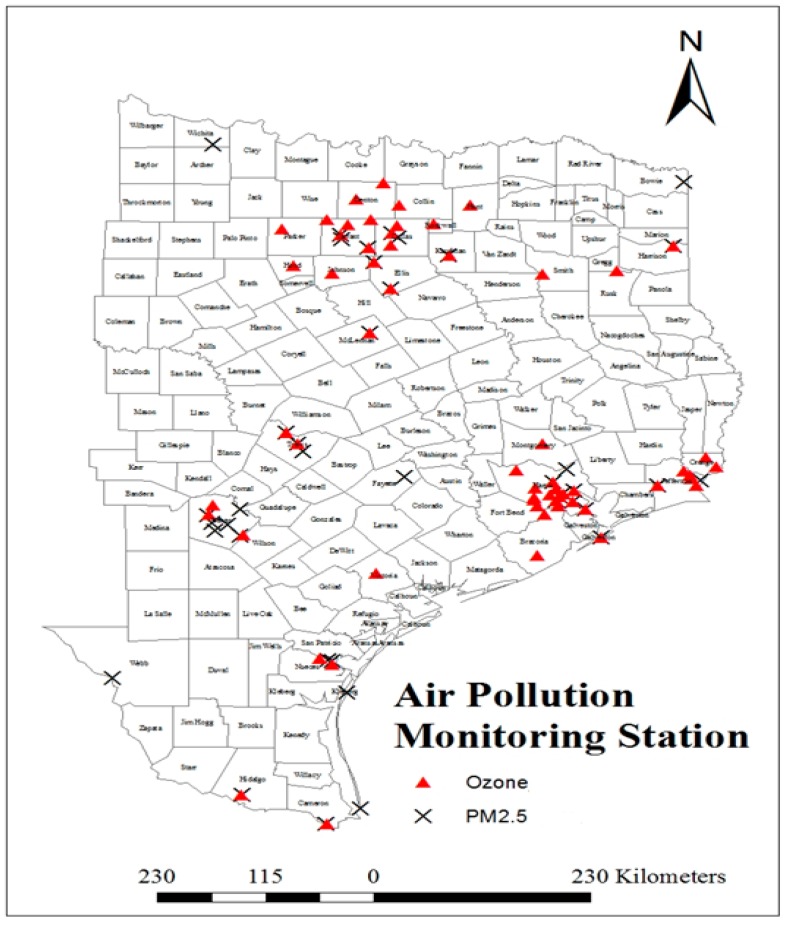
Air pollution monitoring stations in Eastern part of Taxas State.

The present study used the concentrations of two criteria air pollutant parameters (PM_2.5_, and O_3_) for the analyses. Air pollution data routinely collected by U.S. EPA’s Air Quality System (AQS) at 99 U.S. EPA administered monitoring stations (forty for PM_2.5_, and fifty-nine for Ozone) located in different counties of the eastern part of Texas for the year 2010 were used for the study. The air pollution data used in this study were taken from the United States Environmental Protection Agency (U.S. EPA) air quality system data mart [64]. The spatial locations of monitoring stations are represented in Figure 4. The characteristics of the raw data collected from the website are daily average (24 h) concentrations of PM_2.5_, and daily maximum 8 hours average concentrations of ozone. The daily data for each monitoring station were used for determination of quarterly average and annual average concentrations.

#### 3.2.1. Statistical Analyses of Air Pollution Data

The descriptive spatial statistics of the two pollutants (PM_2.5_ and Ozone) are represented in Table 1. In 2010, the minimum quarterly average concentrations of PM_2.5_ in four quarters were 5.5 μg/m^3^, 6.8 μg/m^3^, 8.7 μg/m^3^, and 6.1 μg/m^3^, respectively. In each quarter, the minimum values were observed in the Wichita County except in the third quarter. In the third quarter, the minimum quarterly average concentration was observed in Fayette County. In the same duration, the maximum quarterly average concentrations of PM_2.5_ were 11.7 μg/m^3^, 13.2 μg/m^3^, 14.6 μg/m^3^, and 11.3 μg/m^3^, respectively in 1st, 2nd, 3rd, and 4th quarters. The maximum values were observed in the Harris County in each quarter except the third one. In the third quarter, the maximum concentration was observed in Bowie County.The means of the quarterly average concentrations in four quarters of 2010 were found to be 8.3 μg/m^3^, 10.5 μg/m^3^, 11 μg/m^3^, and 9.1 μg/m^3^, respectively.

Similarly, in the year 2010, the minimum quarterly average concentrations of ozone in four quarters were 30.2 ppb, 33.3 ppb, 25.8 ppb, and 31.9 ppb. These values were observed in the counties of Harris, Hidalgo, Cameron and Harris respectively in 1st, 2nd, 3rd, and 4th quarters.The maximums of quarterly average concentrations of O_3_ in four quarters of 2010 were 42.5 ppb, 49.7 ppb, 49.8 ppb, and 42.8 ppb, respectively. These values were observed in the counties of Orange, Denton, Denton, and Smith respectively in 1st, 2nd, 3rd, and 4th quarters.The mean spatial concentrations in four quarters of 2010 were 37 ppb, 42.2 ppb, 38.4 ppb, and 38.3 ppb, respectively.

The quarterly average data clearly indicate that the mean PM_2.5_ concentration level was highest in third quarter. The mean concentration level of PM_2.5_ was found to be slightly higher than the mean concentration of PM_2.5_ in the second quarter, and was significantly higher than the mean PM_2.5_ concentrations in first and fourth quarters. Similarly, the mean ozone concentration level was found to be highest in the second quarter.

#### 3.2.2. Spatial Analysis of Air Pollution Data

**Figure 5:**
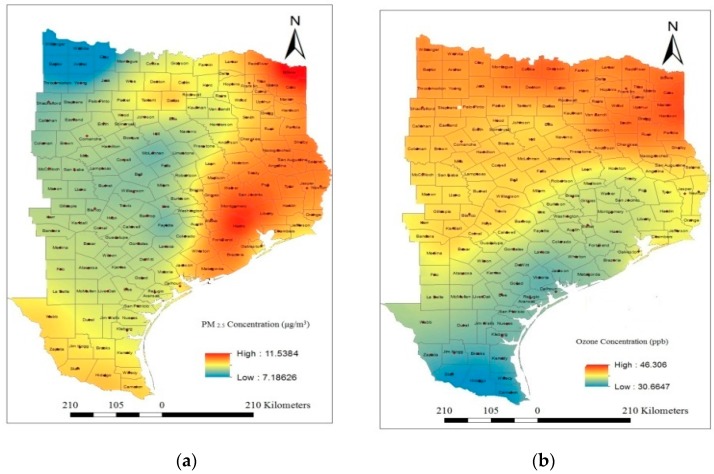
Spatial distribution of air pollutants (**a**) PM_2.5_; (**b**) O_3_.

The spatial locations of each of the selected monitoring stations along with the pollutant concentrations were fed into the GIS system. Interpolation of air pollution data between monitoring stations can be done using geostatistical techniques, such as kriging in its various forms [65] rather than using more complex and empirically based models. Though, there are various types of kriging techniques (ordinary kriging, simple kriging, indicator kriging, universal kriging, and co-kriging) for spatial mapping, the present study used ordinary kriging method for spatial analysis of PM_2.5_ and ozone concentration. The software used for the analysis is Geostatistical Analyst Extension module of ArcGIS version 10.2 [66]. In ordinary kriging interpolation method, a smooth surface is estimated from irregularly spaced data points based on the assumptions that the spatial variation in the feature (O_3_, and PM_2.5_) is homogeneous over the study area. The method interpolates the point data obtained for various monitoring stations in the study area to predict the concentration in each grid cell over a spatial domain. Figure 5a,b depicts the spatial patterns of annual average concentrations of PM_2.5_ and O_3_, respectively, during 2010. A stable type variogram model was used for the prediction of air pollution concentrations at the un-sampled location. The cross-validation results of the model predictions are shown in Table 2. For the cross validation test, the values of mean error (ME), root mean square error (RMSE), average standard error (ASR), mean square error (MSE), and root mean square standardized error (RMSSE) estimated to ascertain the performance of the developed models. A detailed description of the model is provided in the “Geostatistics for Environmental Scientists” book [67].

## 4. Results and Discussion

In recent years, geospatial technology has become increasingly popular in environmental studies. It helps to estimate the individual air pollution via interpolation, and thus minimizing the chances of ecological bias characterized to information loss because of a real data aggregation [68,69,70,71,72,73]. It also helps in deriving the association between the multivariate data. Since the last decade, GIS-based pollution mapping using various interpolation techniques (inverse distance weighted, kriging, and land use regression modeling) has been explored by many researchers for epidemiological studies [74,75,76,77]. Most of the researchers have interpolated mainly the criteria pollutants like PM_2.5_, ozone, SO_2_, and NO_2_ for spatial mapping to determine the exposure level. Asthma data collected from all around the world indicate an increasing trend of asthma morbidity and mortality despite the availability of effective symptomatic treatment. Thus, the problem of asthma has drawn much attention globally. The literature shows that most of the researchers used time-series analyses [41,42,46,78,79,80,81,82,83,84,85,86,87,88,89,90] for understanding the association between air quality and asthma prevalence rather geospatial techniques [74,75,76,77]. In the present study, we demonstrate the application of GIS for analyzing the association between air pollution exposure and ADR, and to visualize major threat areas in the form of maps. The spatial data of two pollutants (PM_2.5_ and ozone) and asthma discharge rate (ADR) in the selected study area were analyzed to understand the association between air pollution and ADR. The association between air pollution exposure and ADR was quantified using the correlation analyses. These are explained in the next section.

### Correlation Analyses

#### Cross Correlation Analyses

The study sought to investigate the association between air pollution exposure and asthma rate for different races (Black, White, and Hispanic), age groups (<5 years, 5–65 years, and >65 years), and genders (Male and Female). Pearson correlation analyses were carried out using the point data represented at the centroid point of each county to understand the association. The concentrations of PM_2.5_ and O_3_ at the centroid point of each county were extracted from the spatial maps as shown in Figure 5a,b respectively. These data were further used in correlation analyses between air pollution level and ADR for different population groups. The ADR at the centroid point of each county is considered to be the same as determined for the individual county.Since, the total numbers of counties in the study area are 157; the numbers of point data derived for the correlation analysis are also 157. The Pearson two-tailed correlation analyses were carried out using SPSS software version 21. The cross-correlation analyses results are presented in Table 3 and indicate that there is a significant variation in correlation coefficient between air pollutants with different populations groups. Though, the results showed a positive association between PM_2.5_ and ADR in each case; the correlations were not statistically significant in most of the cases. Ozone levels showed a negative correlation with asthma rate in most cases. The results also clearly indicate that PM_2.5_ is negatively correlated with the ozone concentration, and hence, it is bound to show a reverse correlation coefficient of ADR with PM_2.5_ and ozone. That is, the result does not mean that ozone concentration reduces the asthma cases. It rather shows that the asthma prevalence is mainly dominated by PM_2.5_ concentrations in the study area.

The study also investigated the association between air pollution exposure level and ADR on a quarterly basis to understand the seasonal effects. The results are represented in Table 4. The results reveal that there is no significant association or relationship between ADR and exposure of air pollutants (PM_2.5_, and O_3_). Though, the results showed a positive correlation between PM_2.5_ and ADR and a negative correlation between ADR and ozone in most of the cases. These correlations were not statistically significant, and can be better explained after considering the local weather conditions.

#### Autocorrelation Analyses

Autocorrelation analyses were carried out to investigate the spatial association between quarterly averages of ADR in different population groups, and conducted in similar ways as cross-correlation analyses. The correlation results for different population groups are presented in Table 5. The correlation analyses results for asthma discharge rate (ADR) clearly indicate that the correlation coefficients among different quarters are statistically significant at 1 percent significance level for each group except Hispanic population. This indicates that the occurrences of asthma cases in different seasons for each group except Hispanic are spatially consistent in nature.

## 5. Limitations of the Study

Although the GIS technology facilitated the research implementation faster and easier, lack of spatial detail and spatial consistency between data sets impeded their full utility. For better and more conclusive results, GIS-based studies need the availability of uniformly distributed pollution and health data. Some data problems and data limitations are encountered with the integration of health data in GIS. Primarily, the interpolation analyses give better pollution level prediction for uniformly distributed spatial data in the study area. However, the pollution monitoring stations in Texas State was not uniformly distributed, and this may lead to some errors in prediction level. Furthermore, there is no state-wide reporting on asthma and, therefore, no centralized asthma database. People suffering from asthma may be seen by a private doctor or may not be seen by any health care provider. This type of cases may not be listed in the asthma database. Again, counties with less than 12 asthma cases are not reported. In addition to above, the asthma data available does not consider the movement of people from one county to another. This study used annual and quarterly average level of pollutants at a particular location as the population's exposure level, but the workplace may not be located in the same county, and this could be biased about an individual’s exposure estimation, which could influence the results. The present study explores the associationsbetween air pollution (PM_2.5_ and ozone)and does not determine causality. There are many environmental variables, including air pollution and some meteorological variables which can influence the prevalence or incidence rate of asthma. Thus, there is a future need for an integrated assessment to evaluate the combined effects of air pollutants and climatic factors on disease rates.

## 6. Conclusions

The study results indicated significant variations in the correlation coefficients between asthma rate and air pollution exposure among different age groups, genders, and races in the residents of East Texas, USA. Furthermore, PM_2.5_ always showed a positive effect in triggering the asthma rate, whereas, the association between ozone and asthma rate did not show a uniform trend. This finding does not mean that ozone reduces the asthma cases, but can be better explained by the fact that asthma prevalence is mainly dominated by PM_2.5_ concentrations in the study area. Auto-correlation results indicated that the occurrences of asthma cases in different seasons for each group except Hispanics, were spatially consistent in nature.That is, few hotspots exist in the study area, where asthma prevalence is more frequent irrespective of seasons. Thus, steps should be taken to protect the most affected populations from further environmental exposure to air pollution, and to control, prevent or reduce the health impacts.

## Figures and Tables

**Table 1:** Descriptive statistics of pollutants and asthma discharge rate in four quarters.

Items	Minimum	Maximum	Mean	Standard Deviation	Minimum	Maximum	Mean	Standard Deviation
	First Quarter of 2010 (Q1)				Second Quarter of 2010 (Q2)			
ADR *	0	10.89	1.92	2.40	0	10.11	1.21	1.80
PM_2.5_ **	5.5	11.7	8.3	1.42	6.8	13.2	10.5	1.30
Ozone ***	30.2	42.5	37.0	2.64	33.3	49.7	42.2	3.85
	Third Quarter of 2010 (Q3)				Fourth Quarter of 2010 (Q4)			
ADR	0	5.46	0.75	1.27	0	10.89	1.35	1.97
PM_2.5_	8.7	14.6	11.0	1.24	6.1	11.3	9.1	1.26
Ozone	25.8	49.8	38.4	5.50	31.9	42.8	38.3	2.28

* ADR is number of asthma-related hospital discharges per 10,000 populations for a specified period of time; ** PM_2.5_ values represented in µg/m^3^; *** Ozone values represented in ppb.

**Table 2:** Prediction Errors of Variogram Model.

Parameter	Type of Variogram Model	ME	RMSE	ASE	MSE	RMSSE
PM_2.5_	Stable	0.099	0.75	0.85	0.06	0.88
Ozone	Stable	−0.090	1.40	1.63	−0.03	0.99

ME: Mean Error; RMS: Root Mean Square Error; ASE: Average Standard Error; MSE: Mean Standard Error; RMSSE: Root Mean Square Standardized Error.

**Table 3:** Correlation analysesresults of annual average data.

Items	Pearson Correlations Coefficients										
	PM_2.5_	Ozone	AR_All	AR_Black	AR_White	AR_Hispanic	AR_Male	AR_Female	AR_LT_5Y	AR_5–65Y	AR_GT_65Y
PM_2.5_	1										
Ozone	−0.100	1									
AR_All	0.244 **	−0.095	1								
AR_Black	0.148	0.082	0.214 **	1							
AR_White	0.181 *	0.222 **	0.733 **	0.258 **	1						
AR_Hispanic	0.039	−0.003	0.134	0.065	−0.061	1					
AR_Male	0.156	−0.007	0.617 **	0.477 **	0.562 **	0.210 **	1				
AR_Female	0.205 **	0.003	0.858 **	0.306 **	0.767 **	0.122	0.655 **	1			
AR_LT_5Y	0.086	−0.037	0.048	0.132	−0.021	0.018	0.149	0.066	1		
AR_5-65Y	0.111	−0.081	0.399 **	0.361 **	0.401 **	0.038	0.550 **	0.497 **	0.074	1	
AR_GT_65Y	0.130	−0.022	0.499 **	0.482 **	0.529 **	0.157 *	0.755 **	0.624 **	0.071	0.461 **	1

**Note:** AR_All—Asthma rate for entire population group; AR_Black—Asthma rate for Black population group; AR_White—Asthma rate for White population group; AR_Hispanic—Asthma rate for Hispanic population group; AR_Male—Asthma rate for Male population group; AR_Female—Asthma rate for Female population group; AR_LT_5Y—Asthma rate for less than 5 years agegroup population; AR_LT_5–65Y—Asthma rate for 5–65 years age group population; AR_GT_65Y—Asthma rate for less greater than 65 years age group population; ** Correlation is significant at the 0.01 level (2-tailed); * Correlation is significant at the 0.05 level (2-tailed).

**Table 4:** Correlation analyses results of quarterly data.

Items	First Quarter (Q1)		Second Quarter (Q2)		Third Quarter (Q3)		Fourth Quarter (Q4)	
	PM_2.5_	Ozone	PM_2.5_	Ozone	PM_2.5_	Ozone	PM_2.5_	Ozone
AR_All	0.104	0.099	0.125	0.057	0.116	−0.009	0.053	0.108
AR_Black	0.012	0.073	0.142	−0.023	0.054	−0.135	0.032	0.026
AR_White	0.108	0.063	0.083	0.031	0.041	0.001	0.039	0.063
AR_Hispanic	0.177 *	−0.042	0.014	0.151	0.147	0.119	0.004	0.018
AR_Male	0.050	−0.027	0.038	0.137	0.088	0.033	−0.045	−0.072
AR_Female	0.031	0.001	0.146	−0.253 **	0.075	0.072	0.090	0.092
AR_LT_5Y	−0.051	0.086	0.070	−0.063	−0.021	−0.103	0.004	0.041
AR_5–65Y	−0.055	−0.054	0.157	−0.162 *	0.004	−0.144	0.173 *	−0.109
AR_GT_65Y	0.077	−0.012	0.115	−0.027	0.024	−0.088	0.104	−0.120

** Correlation is significant at the 0.01 level (2-tailed); * Correlation is significant at the 0.05 level (2-tailed).

**Table 5:** Autocorrelation analyses results.

Entire Population Group									
	Q1		Q2			Q3			Q4
*Q1*	*1*								
*Q2*	*0.684* **		*1*						
*Q3*	*0.568* **		*0.606* **			*1*			
*Q4*	*0.657* **		*0.781* **			*0.503* **			*1*
Female Population Group					Male Population Group				
	Q1	Q2	Q3	Q4		Q1	Q2	Q3	Q4
Q1	*1*				*Q1*	*1*			
Q2	*0.587* **	*1*			*Q2*	*0.773* **	*1*		
Q3	*0.622* **	*0.742* **	*1*		*Q3*	*0.707* **	*0.775* **	*1*	
Q4	*0.547* **	*0.610* **	*0.591* **	*1*	*Q4*	*0.755* **	*0.713* **	*0.749* **	*1*
Black Population Group					White Population Group				
	Q1	Q2	Q3	Q4		Q1	Q2	Q3	Q4
Q1	*1*				Q1	*1*			
Q2	*0.678* **	*1*			Q2	*0.613* **	*1*		
Q3	*0.674* **	*0.667* **	*1*		Q3	*0.583* **	*0.749* **	*1*	
Q4	*0.921* **	*0.722* **	*0.757* **	*1*	Q4	*0.701* **	*0.489* **	*0.435* **	*1*
Hispanic Population Group					<5 Years Age Group				
	Q1	Q2	Q3	Q4		Q1	Q2	Q3	Q4
Q1	*1*				*Q1*	*1*			
Q2	*0.155*	*1*			*Q2*	*0.804* **	*1*		
Q3	*0.968* **	*0.034*	*1*		*Q3*	*0.662* **	*0.636* **	*1*	
Q4	*0.186* *	*0.701* **	*0.044*	*1*	*Q4*	*0.753* **	*0.620* **	*0.752* **	*1*
5–65 Years Age Group					≥ 65 Years Age Group				
	Q1	Q2	Q3	Q4		Q1	Q2	Q3	Q4
Q1	*1*				*Q1*	*1*			
Q2	*0.685* **	*1*			*Q2*	*0.587* **	*1*		
Q3	*0.643* **	*0.879* **	*1*		*Q3*	*0.714* **	*0.763* **	*1*	
Q4	*0.614* **	*0.805* **	*0.815* **	*1*	*Q4*	*0.773* **	*0.725* **	*0.837* **	*1*

** Correlation is significant at the 0.01 level (2-tailed); * Correlation is significant at the 0.05 level (2-tailed).
